# Neurological and mental health consequences of COVID-19: potential implications for well-being and labor force

**DOI:** 10.1093/braincomms/fcab012

**Published:** 2021-02-04

**Authors:** I B Meier, C Vieira Ligo Teixeira, I Tarnanas, F Mirza, L Rajendran

**Affiliations:** 1 Chione GmbH, Studenrain 5, 8122, Binz, Switzerland; 2 Brazilian Institute of Neuroscience and Neurotechnology, Campinas, São Paulo, Brazil; 3 Altoida, Inc, ., 2100 West Loop South, Suite 1450, Houston, TX, 77027, USA; 4 Center for Global and Digital Health (CGDH), Berkeley Suite, 35 Berkeley Square, Mayfair, London, W1J 5BF, UK; 5 UK Dementia Research Institute, King’s College London, London, WC2R 2LS, UK

**Keywords:** COVID-19, cognitive decline, alzheimer’s disease, post-operative cognitive dysfunction, production loss

## Abstract

Recent case studies show that the SARS-CoV-2 infectious disease, COVID-19, is associated with accelerated decline of mental health, in particular, cognition in elderly individuals, but also with neurological and neuropsychiatric illness in young people. Recent studies also show a bidirectional link between COVID-19 and mental health in that people with previous history of psychiatric illness have a higher risk for contracting COVID-19 and that COVID-19 patients display a variety of psychiatric illnesses. Risk factors and the response of the central nervous system to the virus show large overlaps with pathophysiological processes associated with Alzheimer’s disease, delirium, post-operative cognitive dysfunction and acute disseminated encephalomyelitis, all characterized by cognitive impairment. These similarities lead to the hypothesis that the neurological symptoms could arise from neuroinflammation and immune cell dysfunction both in the periphery as well as in the central nervous system and the assumption that long-term consequences of COVID-19 may lead to cognitive impairment in the well-being of the patient and thus in today’s workforce, resulting in large loss of productivity. Therefore, particular attention should be paid to neurological protection during treatment and recovery of COVID-19, while cognitive consequences may require monitoring.

**Abbreviations**

| | |
|---|---|
| ACE2 | Angiotensin-Converting enzyme 2 |
| AD | Alzheimer's disease |
| ADEM | Acute disseminated encephalomyelitis |
| ADRS | Acute respiratory distress syndrome |
| AMPA | α-amino-3-hydroxy-5-methyl-4-isoxazolepropionic acid receptor |
| APOE4 | Apolipoprotein E4 |
| APP | Amyloid Precursor Protein |
| Aβ | Amyloid beta |
| BACE1 | Beta-amyloid cleaving enzyme 1 |
| BBB | Blood brain barrier |
| BNDF | Brain-derived neurotrophic factor |
| COX-2 | Cyclooxygenase-2 |
| CVD | Cerebrovascular disease |
| IL-2, IL-7 | Interleukin |
| ILO | International Labor Organization |
| NMDA | The N-methyl-D-aspartate receptor |
| POCD | Post-operative cognitive dysfunction |
| POD | Postoperative delirium |
| ROS | Reactive oxygen species |
| sHLH | Secondary hemophagocytic lymphohistiocytosis |
| TBI | Traumatic brain injury |
| TMPRSS2 | Transmembrane Serine Protease 2 |
| TNF-α | Tumor Necrosis Factor alpha |
| α-synuclein | Alpha synuclein |

**Introduction**

The outbreak of the new coronavirus (SARS-CoV-2) in late 2019 has since risen to over 76 million confirmed cases today, leading to pneumonia and death in 1.2M of infected individuals globally (*COVID-19 Situation Update Worldwide, as of Week 51 2020*, n.d.). The high transmissibility of the virus quickly led to a world-wide pandemic affecting every age group, and the rapid increase in confirmed cases as well as asymptomatic transmission render the prevention and control of COVID-19 extremely difficult. Increased age (> 40), cardiovascular disease, diabetes, chronic respiratory diseases, and cancer have been identified as risk factors for respiratory symptoms and death (Guan et al., 2020). However, neurological side effects and pathology are starting to become apparent, both in severe cases as well as in cases with light clinical manifestations at any age (Paterson et al., 2020). Therefore, understanding the damage caused by COVID-19 to the brain and underlying mechanisms is of greatest importance, so that treatment of these patients can be timely, effective, and cognitive impairment of elderly, but also the labor force, can be limited and production losses restricted to a minimum.

https://mc.manuscriptcentral.com/braincom

### *Inflammation*

Respiratory failure from acute respiratory distress syndrome (ARDS) is the leading cause of mortality of COVID-19. Research suggests that it may not be the infection *per se* but rather the ensuing hyperinflammation and the cytokine storm causing respiratory dysfunction and fatality (Mudd et al., 2020). Secondary hemophagocytic lymphohistiocytosis (sHLH) is an under-recognized, hyperinflammatory syndrome characterized by hypercytokinemia (Mehta et al., 2020) A cytokine profile resembling sHLH is associated with COVID-19 symptom severity, including increased interleukin (IL)-2, IL-7, interferon-y inducible protein 10, macrophage inflammatory protein 1-alpha, and elevated ferritin, among others, suggesting that mortality might be due to virally driven hyperinflammation (Ruan, Yang, Wang, Jiang, & Song, 2020). Similar hyperinflammation might also take place in the brain. Like most viruses, SARS-CoV-2 has neurotrophic effects, and it is plausible that it enters the central nervous system (CNS) via the olfactory bulb, potentially explaining anosmia as one of the leading symptoms, and may reach the brainstem causing dysfunction and/or death of infected neurons, and that excessive levels of proinflammatory cytokines in the brain result in a cytokine storm with harmful effects in the brain (De Santis, 2020). It is also equally possible that the virus is retrogradely transported from the vagus nerve that innervates the gut, a region where coronavirus has been shown to infect (Naeimi & Ghasemi–Kasman, 2020). Once in the brain, SARS-CoV-2 might infect both neuronal and non-neuronal cells (Crunfli et al., 2020). Activation of microglia could enhance its phagocytic activity towards synaptic structures and result in synapse elimination and consequent cognitive loss (Paolicelli et al., 2017; L. Rajendran & Paolicelli, 2018). Infection in neurons could induce a myriad of pathways including blocking protein synthesis that is essential for memory formation, and impair the endo-lysosomal/ autophagic clearance mechanisms (Yue et al., 2018).

Furthermore, acute disseminated encephalomyelitis (ADEM) affects brain and spinal cord and often results from a minor infection, activating immune cells to attack myelin sheets of neurons and has been identified in the context of COVID-19 (McCuddy et al., 2020). Additional clinical manifestation of brain inflammation due to COVID-19 have been identified as delirium, hallucination, stroke, cognitive impairment, and other neuronal damage in patients ranging 16 to 85 years of age (Paterson et al., 2020).

Concern for long-term cognitive impairment resulting from a SARS-CoV-2 infection stem from research in delirium and post-operative cognitive dysfunction (POCD) in surgery patients. Data from animal and human studies led to assume that peripheral surgical trauma may cause CNS inflammation via the disruption of the blood brain barrier (BBB), leading to disruption of neural activity and resulting in memory dysfunction and cognitive decline (Safavynia & Goldstein, 2018). Peripheral pro-inflammatory cytokines causing inflammation lead to disruption of the BBB permeability via COX-2 upregulation, allowing pro-inflammatory cytokines to enter the CNS. Similar effects may be observed in COVID-19 through infection of the endothelial cells of the BBB through largely overlapping inflammatory markers, notably IL-6, TNF-α, and ROS (see Figure 1).

### *Parallels to Psychiatric illnesses, POCD and AD*

Two studies, one from the UK (Taquet et al., Lancet Psychiatry, 2020) and from Korea (Lee et al., Lancet Psychiatry, 2020) show a bidirectional relationship between COVID-19 and mental health, i.e, elderly people with cognitive disorders have a higher risk for COVID-19 than other adults and COVID-19 infection accelerates brain ageing, mental illness and risk for dementia. There is also a correlation to the apolipoprotein E4 (APOE4) allele: infection of COVID-19 is higher in ApoE4 carriers who are vulnerable to Alzheimer's disease (AD). The UK study performed in 451,367 patients, those with the ApoE e4e4 gene variant are 2.3 to 4 times more prone to be infected with and test positive for COVID-19 than others. According to the Center of Disease Control (CDC), more than 31,000 people with Alzheimer's disease died during the pandemic and 1700 in New York State, a 21% increase from the past years showing how COVID-19 could disproportionally affect AD patients (www.cdc.org).

While no longitudinal data is available on how COVID-19 affects elderly people's cognition, mechanisms in the inflammatory response in AD that resemble processes caused by COVID-19 are leading to the assumption that elderly patients' cognition will be particularly affected by this pandemic (Hampshire et al., 2020). As mentioned, SARS-CoV-2 could directly act on the cells of the central nervous system and impair synapse function. On the one hand, attempts to avoid the contamination through SARS-CoV-2 such as social distancing and isolation increase the risk of depression and other mental disorders, contributing to cognitive decline and potentially increasing the risk of developing Alzheimer's disease (Fontes et al., 2020; Ownby et al., 2006) through hypothalamic-pituitary-adrenalin axis dysregulation, hippocampal atrophy, inflammatory changes, cerebrovascular disease and increased amyloid deposition (Dafsari & Jessen, 2020). One potential disease mechanism could be chronic inflammation leading to reduced synaptic availability and release of monoamines in the brain. Alternatively, loss of astroglia and chronic microglial activation have been associated with greater duration of depression and is considered to be involved in the progression of neurodegenerative diseases (Dafsari & Jessen, 2020). Depression has also been associated with a reduction noradrenalin and serotonin, altering amyloid clearance, and cerebrovascular disease, an independent risk factor for AD (Eriksson et al., 2010; graphical abstract).

Changes in lifestyle patterns such as exercise, a healthy diet, and staying cognitively active are known to be protective against AD (Ngandu et al., 2015) and are diminished given the current circumstances of the pandemic, exacerbating risks of dementia even further. A recent study (Xie, Campbell, & Zhang, 2020) suggested that compliance to social distancing and practice of hygiene were already dependent on the working memory of individuals, highlighting a complex relationship between cognitive function and infection with SARS-CoV-2.

On the other hand, contraction of the disease in elderly patients has been observed to impact their cognition (Heneka, Golenbock, Latz, Morgan, & Brown, 2020). While long-term studies are needed to prove clinical observations, a series of similarities in the disease pathogenesis require more extensive investigation. The 'cytokine storm' of proinflammatory cytokines such as IL-6 and TNF-α observed in COVID-19, in addition to Aβ and phosphorylated tau, resemble processes of the pathogenesis of AD and inflammatory response observed post-surgery. The loss of smell, that appears to be transient in COVID-19 but appears early and irreversibly in AD, may hint at another common pathway: perineural spaces that encompass olfactory nerves and the nasal lymphatics are important for CSF drainage (Sokołowski et al., 2018). SARS-CoV-2 may directly infect olfactory sensory neurons in the epithelium and then transported into the CNS through the olfactory nerve (Li et al., 2020).

It is equally possible that SARS-CoV-2 does not cross the blood brain barrier or gain entry to the CNS, however, through peripheral inflammation as witnessed in the case of HIV-associated neurodegeneration (Hu et al., 2016). In the case of brain injury caused by stroke or neuroinflammation through viruses, astrocytes have been shown to release exosomes or extracellular vesicles that prod the release of cytokines that enable infiltration of immune cells into the brain causing neuronal damage (Dickens et al., 2017). Similar mechanisms could occur in the case of SARS-CoV-2 and cause both structural (synaptic elimination) as well as cognitive damage (synaptic dysfunction).

Genetic variants also seem to influence how COVID-19 impacts patients. Elderly people with apolipoprotein E (ApoE4), known to pose an increased risk to develop AD, are known to undergo a more severe course of COVID-19 than people that are non-carriers, and ApoE e4e4 homozygotes are more likely to be COVID-positive compared to e3e3 homozygotes (Kuo et al., 2020). ApoE4 additionally brings an elevated risk of cardiovascular disease such as heart disease and stroke, moderates macrophage pro-/anti-inflammatory phenotypes, and is associated with more microbleeds, which are known to disrupt the BBB. Surprising MRI findings of cerebral microbleeds in some of the COVID patients propose an additional parallel between COVID-19 and AD: In AD, cerebral microbleeds appear to reflect primarily injury associated with hypertensive vasculopathy when occurring in deep brain structures, increasing the risk of stroke, while lobar microbleeds primarily reflect cerebral amyloid angiopathy but could also be impacted by ischemia and are known to contribute to cognitive decline (Meier et al., 2014). In the context of COVID-19, microbleeds are assumed to occur due to endothelial dysfunction related to viral binding to the ACE-2 receptors expressed on endothelial cells and may pose a risk factor for stroke, however, further studies are required to establish this relationship (Paterson et al., 2020). Nonetheless, microbleeds are known to disturb vascular reactivity and cerebral blood flow, further disrupting normal brain functioning, and are associated with BBB dysfunction, yet the causal relationship is unclear.

At the cellular level, both the pathogenic aspects of the viral entry and the production of the amyloid peptide of AD are remarkably similar. In the case of AD, the substrate protein APP (Amyloid Precursor Protein) and its protease BACE1 (beta-amyloid cleaving enzyme 1) or beta-secretase are both internalized through clathrin mediated and cholesterol/raft mediated pathways and in the early endosomes, where the pH is conducive for the proteolytic activity of BACE1, APP is cleaved by BACE1 and then subsequently by γ-secretase to release the amyloid peptide in the early endosomes (Zhang & Song, 2013). Similarly, the SARS-CoV-2's spike protein is first proteolytically cleaved by Furin, a protease that releases the spike fusion peptide, thus preparing the pre-activation phase of facilitating the virus entry through its receptor, Angiotensin-Converting enzyme 2 (ACE2) (Letko, Marzi, & Munster, 2020) and Transmembrane Serine Protease 2 (TMPRSS2) (Hoffmann et al., 2020). Interestingly, SARS-CoV-2 binds ACE2 at the clathrin coated pits and uses endocytic mechanisms similar to that of APP/BACE1 of AD (Bayati, Kumar, Francis, & McPherson, 2020; Lawrence Rajendran et al., 2006; Lawrence Rajendran et al., 2008) and exit through lysosomes (Rajendran & Annaert, 2012; Ghosh et al., 2020). The consequences of the endosomal or lysosomal entry into cytosol could have serious implications that could lead to dysregulated lysosomal clearance and autophagy that could cause the impaired clearance and thus the accumulation of misfolded proteins such as Aβ, Tau, or α-synuclein, proteins implicated in AD or Parkinson's disease. These misfolded proteins have been shown to cause synaptic inhibition and also synapse loss (Hong et al, Science, 2016). Viral infection of neurons could also induce impairment of recycling of receptors such as NMDA or AMPA that could lead to significant inhibition in synaptic transmission that could lead to various neuropsychiatric disorders depending on which neuronal or non-neuronal cellular populations that are infected. Infection of microglia or astrocytes could also lead to impaired cytokine secretion of phagocytic clearance of neuronal misfolded proteins thus affecting several homeostatic functions in the brain.

Taken together, striking similarities in inflammatory response, cytokine storm, vascular risk factors, small vessel cerebrovascular disease, the disruption of the BBB, and cognitive impairment as a consequence seem to form a common denominator of post-operative delirium (POD)/POCD and AD with COVID-19. From case studies we know that both elderly and younger patients can suffer from neuronal consequences of COVID-19, whether or not they underwent a severe disease course Paterson et al., 2020. The long-term consequences remain unknown, but are to be taken seriously, as they may not only put elderly people at increased risk for dementia but may also cognitively impair a much younger population.

***Hypoxia***

Patients with COVID-19 regularly suffer from severe hypoxia and viremia (Guo et al., 2020), potentially causing infectious toxic encephalopathy, a type of reversible brain dysfunction syndrome (Alomari et al., 2020). Almost 40% of COVID-19 patients develop headache, disturbed consciousness and other brain dysfunction symptoms and acute cerebrovascular disease (Wu et al., 2020). When a virus enters lung tissue cells, it causes diffuse inflammation, edema, and may cause hypoxia in the CNS, causing subsequent nervous system damage. Resulting increasing anaerobic metabolism in the mitochondria of brain cells can lead to cerebral vasodilation, swelling of brain cells and obstruction of cerebral blood flow. Consequences include headache due to ischemia, acute ischemic stroke, but also cerebral circulation disorders that may irreversibly affect the brain and cognition.

In surgery patients, postoperative hypoxia is a large contributing factor to cognitive impairment (Browne, Halligan, Wade, & Taggart, 2003). Hypoxia is known to negatively affect cerebral blood flow and blood oxygenation, while cerebral blood flow itself is associated with the regional deposition of beta-amyloid (Aβ), a hallmark pathological marker of Alzheimer's disease. Mild chronic cerebral hypoperfusion may create a metabolically dysregulated microenvironment triggering entry and accumulation of peripherally applied amyloid peptides. Amyloid deposit in brain arteries may induce hypoperfusion itself via decreased vasodilation or impaired clearance of pathology (Meier et al., 2020). While the causal relationship between cerebral perfusion and amyloid is unclear, cognitive impairment is a known association with both. It is hence possible that hypoxia in COVID-19 patients may lead to long term cerebral perfusion changes and potentially increase the deposit of brain pathology, leading to cognitive impairment and increasing risk for AD.

***Production loss due to potential neurological consequences of COVID-19***

According to the ILO, global working hours declined by 4.5 per cent (ca. 130 million full-time jobs in a 48 hour working week) in the first quarter of 2020, compared to pre-crisis situation, and were up to 10.5% lower in the second quarter, while lower-middle-income countries are expected to register the highest rate of hours lost (12.5%) (ILO Report, April 29, 2020). Urgent policies are needed to support workers and businesses. However, when looking at the employed workforce, occupational risks affect different work groups differently. In a study assessing almost 10'000 healthcare employees in the UK, 11% of staff had evidence of COVID-19 (COVID-19: Differential occupational risks to healthcare workers from SARS-CoV-2). Increased rates of COVID-19 were found in staff working in COVID-19 facing areas (21.2% vs 8.2% elsewhere). When it comes to hospitalizations, we know from a report from Harvard University that about one third of COVID-19 patients in intensive care experienced cognitive impairment equivalent to people who suffered a traumatic brain injury (TBI), including memory gaps, attention issues, and problems with simple tasks (*The Hidden Long-Term Cognitive Effects of COVID-19 - Harvard Health Blog - Harvard Health Publishing*). In an online study from Imperial College London that tested almost 85'000 patients (Hampshire et al, 2020), the subgroup that was hospitalized with ventilator showed cognitive impairment equivalent to a 10-year decline in global performance between the ages of 20 to 70. Impairments affected multiple domains, particularly semantic problem solving and visual attention. Recovering from COVID-19 brought along pronounced problems in executive functions, which is also a domain impacted early in AD, although this could generally be due to particularly sensitive executive functioning assessments. The most alarming finding, however, is that young adults as early as in their 20ies showed such large cognitive impairments, and that these alterations may be persistent, causing a large economic burden in healthcare costs and more importantly, also production loss.

Gauging potential production loss due to neurological consequences of COVID-19 in the labor force at this stage is unpredictable. More data is required to identify the incidence rate of neurological disorders related to the SARS-CoV-2 infection in younger people and whether effects are reversible. Furthermore, it will be necessary to follow the cases that suffered from neurological disorders over multiple years in order to establish whether any premature cognitive impairment appears related to the infection. However, when looking at data from traumatic brain injury, which frequently appears in younger individuals and leads to cognitive decline and production loss, median production losses due to early retirement are estimated to be around 1.35M USD per patient (Tuominen, Joelsson, & Tenovuo, 2012). A 2010 estimate of production loss due to mental and neurological disorders came out to 3-5M USD (Bloom, 2011). These estimates do not include treatment costs, nor are they directly applicable to the case of COVID-19, but they hint at the potential heavy societal and economic burden imposed by the virus, independent of the mental health disorders such as anxiety and depression arising through COVID precautionary measures.

Lockdown, social distancing, isolation, and working from home, or traumatic events such as death of friends and relatives and not being able to be close to them certainly has impacted each and everyone's mental state and caused surges in depression, anxiety, restlessness, stress, and other psychiatric symptoms, at least during the initial phases of the COVID-19 pandemic (Ritchie et al., 2020). However, hope remains that mental health will become less stigmatized and more openly talked about and acted upon in the years to come. Additionally, it will be crucial for employers to be aware, vigilant, tolerant, and supportive in providing help. Employment laws such as the non-discrimination law against mental health, to which cognitive impairment belongs, may be an initial and much needed step to protect employees. Additional support such as occupational therapy, physical exercise, and cognitive training could be beneficial for patients having suffered from cognitive impairment due to COVID-19, however, interventional studies are required to reach a conclusion about the most effective treatments and interventions, while prevention remains key.

## Conclusions

Case studies showing neuronal death in the context of COVID-19 in young people with a light course of the disease are proposing a somber scenario with potential long-term consequences of cognitive impairment, given that it may affect both older and younger people. Mental and neurological disorders independent of COVID-19 consequences cause multiple trillion losses in lost output. Under the assumption that COVID-19 may affect and eventually cognitively impair the current labor force, massive financial damage would extend manifold to multiple trillions through production loss, aside from putting an additional strain on the healthcare system and public health costs. Hope remains that a treatment for COVID-19 will be found soon, and should the disease mechanisms between COVID-19, POD/POCD and AD turn out to be as similar as currently assumed, a desirable side effect of these medications may benefit elderly patients post-surgery and with dementia, while keeping our labor force cognitively healthy.

## Data availability statement

Data sharing is not applicable to this article as no new data were created or analyzed in this study.

## Competing interests

None of the authors have competing interests to declare

## Funding

No funding or grants were received for this manuscript.

**Figure legend**

**Figure 1. Schematic similarities of the neuroinflammatory and -degenerative pathways between COVID-19 and Alzheimer's disease/post-operative cognitive dysfunction.** Joint risk factors increase and/or accelerate the disease severity, while a cascade of molecular and biochemical processes is initiated. Illustration not exhaustive. More details about underlying processes can be found in Dafsari & colleagues (2020).

https://mc.manuscriptcentral.com/braincom

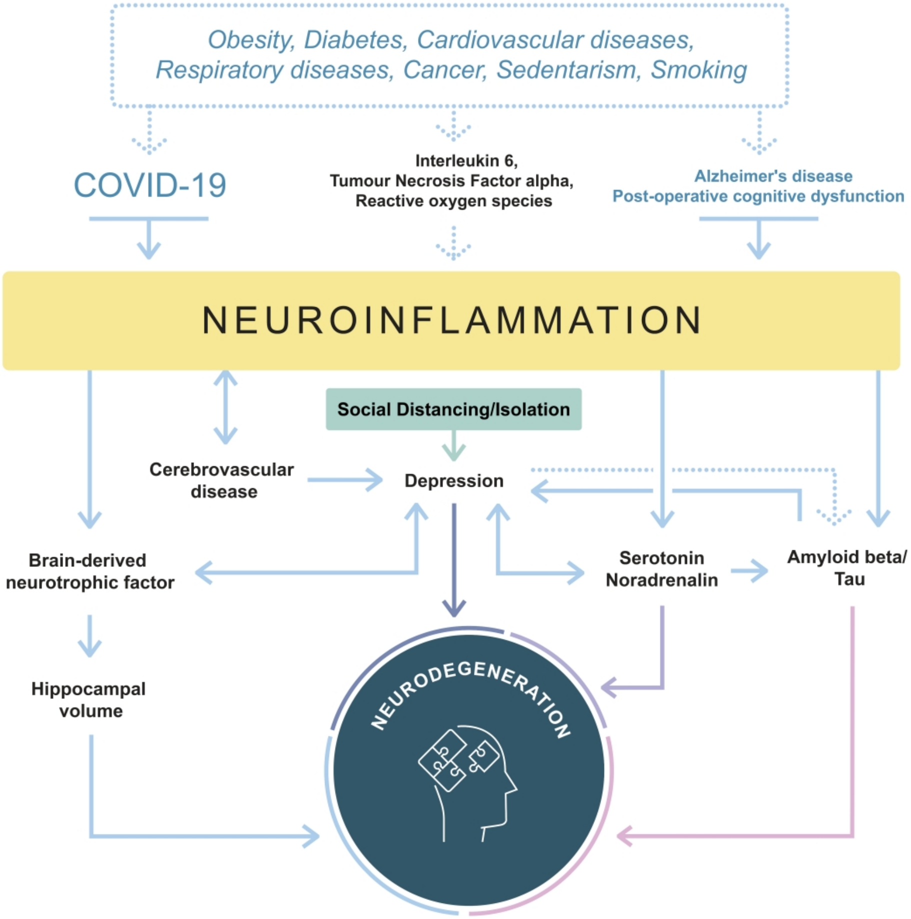

Figure 1. Schematic similarities of the neuroinflammatory and -degenerative pathways between COVID-19 and Alzheimer's disease/post-operative cognitive dysfunction. Joint risk factors increase and/or accelerate the disease severity, while a cascade of molecular and biochemical processes is initiated. Illustration not exhaustive. More details about underlying processes can be found in Dafsari & colleagues (2020).

1387x1387mm (96 x 96 DPI)

Abbreviated summary

Meier et al. contemplated the similarities of risk factors, neuroinflammatory processes, and potential neurological consequences between COVID-19 and Alzheimer's disease/post-operative cognitive dysfunction. The observations yield the assumption that long-term consequences of COVID-19 may lead to cognitive impairment even in young individuals, affecting today's workforce and eventually resulting in production loss.

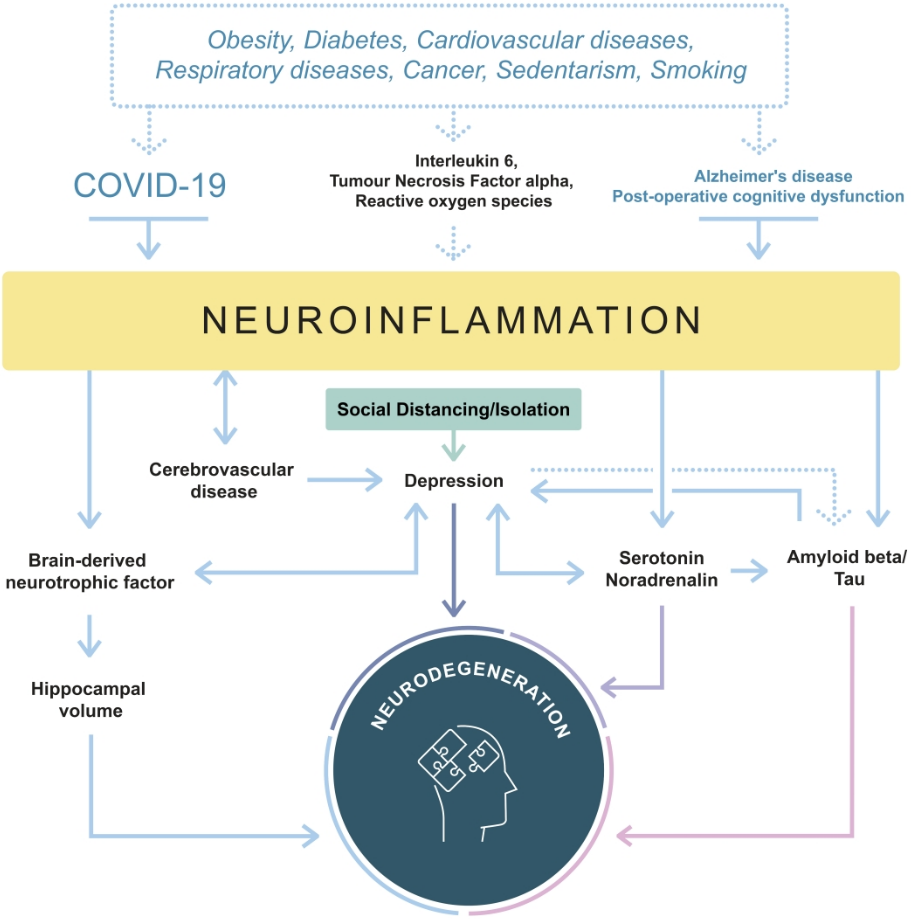

Graphical abstract

1387x1387mm (96 x 96 DPI)
